# Natural Killer T Cells in Cancer Immunotherapy

**DOI:** 10.3389/fimmu.2017.01178

**Published:** 2017-09-22

**Authors:** Shiny Nair, Madhav V. Dhodapkar

**Affiliations:** ^1^Yale University, New Haven, CT, United States

**Keywords:** natural killer T, glycolipid antigens, CD1d, dendritic cells, innate immunity

## Abstract

Natural killer T (NKT) cells are specialized CD1d-restricted T cells that recognize lipid antigens. Following stimulation, NKT cells lead to downstream activation of both innate and adaptive immune cells in the tumor microenvironment. This has impelled the development of NKT cell-targeted immunotherapies for treating cancer. In this review, we provide a brief overview of the stimulatory and regulatory functions of NKT cells in tumor immunity as well as highlight preclinical and clinical studies based on NKT cells. Finally, we discuss future perspectives to better harness the potential of NKT cells for cancer therapy.

## Introduction

Both innate and adaptive immune systems respond to tumor cells and participate in immune-surveillance against tumor (1). Defined immune interactions in the context of cancer include recognition of tumor-associated antigens or cues by innate cell populations such as antigen-presenting cells (APCs) [macrophages and dendritic cells (DCs), neutrophils, and natural killer (NK) cells (2)]. Innate immune cells rely on germline encoded pattern recognition receptors to recognize and elicit prompt response against cancer-associated danger signals, and also augment components of the adaptive immune system, composed of antigen-specific B and T cells (1). One of the key players that link the innate and adaptive immune systems is the natural killer T (NKT) cells (3–5). NKT cells are innate-like T lymphocytes that possess ability to quickly respond to antigenic stimulation and rapidly produce copious amounts of cytokines and chemokines (6). This rapid effect can modulate both innate and adaptive immunity and is important in influencing host immune responses to cancer (7).

Natural killer T cells are a heterogeneous subset of specialized T cells (8). These cells exhibit innate cell-like feature of quick response to antigenic exposure in combination with adaptive cell’s precision of antigenic recognition and diverse effector responses (9). Like conventional T cells, NKT cells undergo thymic development and selection and possess T cell receptor (TCR) to recognize antigens (10). However, unlike conventional T cells, TCR expressed by NKT cells recognize lipid antigens presented by the conserved and non-polymorphic MHC class 1 like molecule CD1d (11). In addition to TCRs, NKT cells also possess receptors for cytokines such as IL-12, IL-18, IL-25, and IL-23 similar to innate cells such as NK and innate lymphoid cells (12). These cytokine receptors can be activated by steady state expression of these inflammatory cytokines even in the absence of TCR signals. Thus, NKT cells can amalgamate signals from both TCR-mediated stimulations and inflammatory cytokines to manifest prompt release of an array of cytokines (13). These cytokines can in turn modulate different immune cells present in the tumor microenvironment (TME) thus influencing host immune responses to cancer. Their predominant tissue localization and ability to sense cancer-mediated changes in host lipid metabolism or breach in tissue integrity *via* recognition of endogenous lipids, makes NKT cells an ideal candidate for cancer immunotherapy (14).

## Type I NKT Cells

Broadly, CD1d-restricted NKT cells can be divided into two main subsets based on their TCR diversity and antigen specificities. Type I (invariant) NKT cells, so named because of their limited TCR repertoire, express a semi-invariant TCR (iTCR) α chain (Vα14-Jα18 in mice, Vα24-Jα18 in humans) paired with a heterogeneous Vβ chain repertoire (V β 2,7 or 8.2 in mice and V β 11 in humans) (8, 9). The prototypic antigen for type I NKT cells is galactosylceramide (α-GalCer or KRN 7000), which was isolated from a marine sponge as part of an antitumor screen (15). α-GalCer is a potent activator of type I NKT cells, inducing them to release large amounts of interferon-γ (IFN-γ), which helps activate both CD8^+^ T cells and APCs (16, 17). The primary techniques used to study type I NKT cells include staining and identification of type I NKT cells using CD1d-loaded α-GalCer tetramers, administering α-GalCer to activate and study the functions of type I NKT cells and finally using CD1d deficient mice (that lack both type I and type II NKT) or Jα18-deficient mice (lacking only type I NKT) (10). Recent published study reported that Jα18-deficient mice in addition to having deletion in the *Traj18* gene segment (essential for type I NKT cell development), also exhibited overall lower TCR repertoire caused by influence of the transgene on rearrangements of several Jα segments upstream *Traj18*, complicating interpretations of data obtained from the Jα18-deficient mice (18). To overcome this drawback, a new strain of Jα18-deficient mice lacking type I NKT cells while maintaining the overall TCR repertoire has been generated, which should facilitate future studies on type I NKT cells (19). Type I NKT cells can be further subdivided based on the surface expression of CD4 and CD8 into CD4^+^ and CD4^−^CD8^−^ (DN) subsets and a small fraction of CD8^+^ cells found in humans (6, 20–24). Type I NKT cells are present in different tissues in both mice and humans but at higher frequency in mice (25, 26). Two very unique characteristics of type I NKT cells are that they possess dual reactivity to both self and foreign lipids, and that even at steady state type I NKT cell have an activated/memory phenotype (6, 27, 28). Functionally distinct subsets of NKT cells analogous to Th1, Th2, Th17, and TFH subsets of conventional T cells have been described. These subsets express the corresponding cytokines, transcription factors and surface markers of their conventional T cell counterparts (29–31). Type I NKT cells have a unique developmental program that is regulated by a number of transcription factors (32). Transcriptionally, one of the key regulators of type I NKT cell development and activated memory phenotype is the transcription factor promyelocytic leukemia zinc finger (PLZF). In fact, PLZF deficient mice show profound deficiency of type I NKT cells and cytokine production (33, 34). Other transcription factors that are known to impact type I NKT cell differentiation are c-Myc (35, 36), RORγt (37), c-Myb (38), Elf-1 (39), and Runx1 (40). Furthermore, transcription factors that control conventional T cell differentiation such as Th1 lineage specific transcription factor T-bet and Th2 specific transcription factor GATA-3 can also affect type I NKT cell development (41–43). Aside from transcription factors, SLAM-associated protein (SAP) signaling pathway can also selectively control expansion and differentiation of type I NKT cell (44, 45). Type I NKT cells have been shown to respond to both self and foreign α and β linked glycosphingolipids (GSL), ceramides, and phospholipids (46). Type I NKT cells have been reported to mostly aid in mounting an effective immune response against tumor (3, 5, 47–49).

## Type II NKT Cells

Type II NKT cells also called diverse or variant NKT cells, are CD1d-restricted T cells that express more diverse alpha-beta TCRs and do not recognize α-GalCer (50). Type II NKT cells are major subset in humans with higher frequency as compared to type I NKT cells (51). Due to absence of specific markers and agonistic antigens to identify all type II NKT cells, characterization of these cells has been challenging. Different methodologies employed to characterize type II NKT cells include, comparing immune responses between Jα18^−/−^ (lacking only type I NKT) and CD1d^−/−^ (lacking both type I and type II NKT) mice, using 24 αβ TCR transgenic mice (that overexpresses Vα3.2/Vβ9 TCR from type II NKT cell hybridoma VIII24), using a Jα18-deficient IL-4 reporter mouse model, staining with antigen-loaded CD1d tetramer and asses binding to type II NKT hybridomas [reviewed in Ref. (46)]. The first major antigen identified for self-glycolipid reactive type II NKT cells in mice was myelin derived glycolipid sulfatide (25, 26, 52). Subsequently, sulfatide and lysosulfatide reactive CD1d-restricted human type II NKT cells have been reported (53, 54). Sulfatide specific type II NKT cells predominantly exhibit an oligoclonal TCR repertoire (V α 3/V α 1-J α 7/J α 9 and V β 8.1/V β 3.1-J β 2.7) (25). Other self-glycolipids such as β GlcCer and β GalCer have been shown to activate murine type II NKT cells (55–57). Our group recently reported that two major sphingolipids accumulated in Gaucher disease (GD), β-glucosylceramide (β GlcCer) and its deacylated product glucosylsphingosine, are recognized by murine and human type II NKT cells (57). In an earlier study, we have also shown that lysophosphatidylcholine (LPC), lysophospholipid markedly upregulated in myeloma patients was an antigen for human type II NKT cells (58). Type II NKT cells can be distinguished from type I NKT cells by their predominance in humans versus mice, TCR binding and distinct antigen specificities (59). Crystal structures of type II NKT TCR-sulfatide/CD1d complex and type I NKT TCR-α-GalCer/CD1d complex provided insights into the mechanisms by which NKT TCRs recognize antigen (60). The type I NKT TCR was found to bind α-GalCer/CD1d complex in a rigid, parallel configuration mainly involving the α-chain. The key residues within the CDR2β, CDR3α, and CDR1α loops of the semi-iTCR of type I NKT cells were determined to be involved in the detection of the α-GalCer/CD1d complex (61). On the other hand, type II NKT TCRs contact their ligands primarily *via* their CDR3β loop rather than CDR3 α loops in an antiparallel fashion very similar to binding observed in some of the conventional MHC-restricted T cells (62). Ternary structure of sulfatide-reactive TCR molecules revealed that CDR3 α loop primarily contacted CD1d and the CDR3β determined the specificity of sulfatide antigen (63). The flexibility in binding of type II NKT TCR to its antigens akin to TCR–peptide–MHC complex resonates with its greater TCR diversity and ability to respond to wide range of ligands. However, despite striking difference between the two subsets, similarities among the two subsets have also been reported. For example, both type I and type II NKT cells are autoreactive and depend on the transcriptional regulator PLZF and SAP for their development (55, 64, 65). Although, many type II NKT cells seem to have activated/memory phenotype like type I NKT cells, in other studies including ours, a subset of type II NKT cells also displayed naïve T cell phenotype (CD45RA^+^, CD45RO^−^, CD62^high^, and CD69^−/low^) (66, 67). Type II NKT cell is activated mainly by TCR signaling following recognition of lipid/CD1d complex (56, 68) independent of either TLR signaling or presence of IL-12 (65, 69).

In tumor and autoimmune disease models, type II NKT cells are typically associated with immunosuppression (70–72).

## How Do NKT Cell Target Tumor Cells?

**Figure 1:**
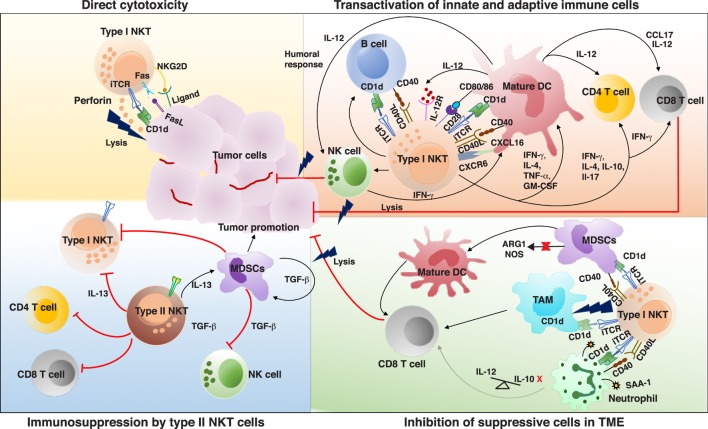
Interactions and cross talk between different subsets of natural killer T (NKT) cells and other immune cells in tumor microenvironment (TME). Antigenic activated type I NKT cells can promote antitumor immunity by directly killing tumor cells in a CD1d-dependent and -independent mechanism. Type I NKT cells can recognize self or foreign lipid antigens presented by different CD1d-expressing antigen-presenting cells (APCs) in TME such as dendritic cells (DCs), TAMs, B cells, and neutrophils. On activation type I NKT cells can produce various Th1 and Th2 cytokines leading to reciprocal activation and or modulation of the APCs as well as other effector lymphocytes. Major type I NKT cytokine that helps activate DCs and CD8^+^ T cells is interferon-γ (IFN-γ). Type I NKT cells and DCs reciprocally activate each other *via* CD1d-TCR/lipid antigen and CD40–CD40L interactions. IL-12 produced by type I NKT cell matured DCs stimulates natural killer (NK), NKT, and MHC-restricted T cells to produce more IFN-γ which can secondarily activate other antitumor-promoting effector lymphocytes. Mature DCs derived factors as well as costimulatory receptors can activate CD8^+^ T cells to promote adaptive immunity. Type I NKT cells enhance tumor immunity by subduing the actions of tumor supporting cells such as TAMs, MDSCs, and suppressive neutrophils. In some instances, type II NKT cells have been shown to suppress the activation of type I NKT cells, T cells, NK cells and enhance development of tumor-associated MDSCs, aiding in tumor growth. iTCR, invariant TCR; IL-12, interleukin 12; IL-12R, IL-12 receptor; CXCL16, chemokine ligand 16; CXCR6, chemokine receptor 6; MDSCs, myeloid-derived suppressor cell; TAM, tumor-associated macrophages; ARG1, arginase 1; NOS, nitrous oxide synthase; SAA-1, serum amyloid A1; TCR, T cell receptor.

Several clues exist attributing a significant role of type I NKT cells in mediating protective immune response against tumors. Decreased frequency and function of type I NKT cells in the peripheral blood of different cancer patients is suggestive of their role in effective antitumor immunity (73–78). Increased frequency of peripheral blood type I NKT cells in cancer patients predicts a more favorable response to therapy (79, 80). Furthermore, recent studies found an association between number of tumor-infiltrating NKTs with better clinical outcome (79, 81). Notably, α-GalCer, the prototypic NKT ligand, was first discovered in a screen for antitumor agents (82). Many studies using genetic knockouts and murine models of tumor have been useful to discern the role of NKT cells in malignancy (83, 84). Type I NKT cells can lead to effective antitumor immunity by three mechanisms: (a) direct tumor lysis, (b) recruitment and activation of other innate and adaptive immune cells by initiating Th1 cytokine cascade, and (c) regulating immunosuppressive cells in TME (Figure 1).

## Direct Cytotoxicity Against Tumor Cells

Natural killer T cells can eliminate CD1d-expressing transformed cells by direct cytolysis using either perforin (85, 86), granzyme B, Fas ligand (FasL) (87, 88), or TNF-α-mediated cytotoxic pathways (89). Tumor cells expressing CD1d are mainly of myelomonocytic and B-cell lineages origin (90), and very few solid tumors have also been found to be CD1d-positive (91–95). Surface expression of CD1d on tumor cells is assumed to directly correlate with NKT cell-mediated cytotoxicity (96). With higher expression of CD1d, resulting in higher tumor cell lysis and thereby lower metastasis rates (92, 97), while lack of CD1d expression in tumors leads to their escape from recognition by NKT cells, and tumor progression in some models (90, 98, 99). These studies postulate that loss or downregulation of surface expression of CD1d favors tumor survival and permits tumor escape from NKT cell-mediated immunosurveillance. This concept is further strengthened by observations that downregulation of CD1d in human breast cancer and multiple Myeloma correlated with increased metastatic potential and disease progression (92, 99). Similarly, downregulation of CD1d by human papillomavirus in infected cervical epithelial cells was linked to their progression to cervical carcinoma (100). Another means by which tumor cells escape NKT cell-mediated antitumor response was shown in a mouse model of lymphoma, where shedding of tumor-associated glycolipids was shown to inhibit CD1-mediated presentation to NKT cells (101). Interestingly, in chronic lymphocytic leukemia (CLL), CD1d expression was found to increase during disease progression, counteracting the suggested role of CD1d as an anti-survival factor in cancer (102, 103). However, a recent study has shown that higher CD1d expression on CLL cells associated with disease progression actually led to impairment in both function and numbers of type I NKT cells (104). CD1d independent cytotoxic effect of NKT cells on various hematopoietic tumor cell lines have also been reported (98, 105, 106). Although, the mechanisms or tumor specific CD1d—glycolipid complex that helps NKT cells recognize and kill only CD1d-positive tumor cells and not normal cells is still enigmatic. Membrane glycolipids especially GSL such as globotriaosyl-ceramide (Gb3Cer/CD77), gangliosides (GD2, GD3, and GM2) have been shown to be overexpressed and altered in a range of cancers compared to normal tissue (107, 108). Shedding of some of the gangliosides and GSL into the TME have also been reported. Recognition of these overexpressed GSL and gangliosides on the surface of tumor cells may lead to differential recognition and killing of tumor cells by NKT cells.

## Cytokine-Mediated Modulations of Effector Cells

In addition to direct tumor lysis, type I NKT cells can activate and recruit both innate and adaptive immune cells, such as DCs, NK cells, B cells, and T cells through rapid secretion of cytokines on activation (109). This is underscored by the observed increase in NK cells, CD8^+^ T cells and macrophages among tumor-infiltrating leukocytes brought about by α-GalCer injection (110). Owing to partially activated state and the presence of preformed cytosolic mRNA for various cytokines, type I NKT cells can rapidly produce broad spectrum of Th1 and Th2 cytokines on activation (111–113). The nature and magnitude of the type I NKT cell cytokine response is contingent on a number of variables that include the glycolipid antigen, subsets of NKT, and tissue location. For example, while α-GalCer-activated type I NKT cell primarily elicits an IFN-γ, a synthetic analog of α-GalCer with a truncated lipid chain OCH elicits majorly elicits IL-4 production (114). Further, DN liver subset of type I NKT was found to confer protection as compared to CD4^+^ liver subset or IL-4 inducing thymic type I NKT cells in MCA-induced fibrosarcoma model (115). Type I NKT cells play a crucial role in induction of early immune responses to tumor by influencing DC maturation (116). Mostly DCs found in TME are immature and inept at activating specific T cells (117). Maturation and differentiation of DCs is important in shaping the magnitude and polarization of T cell-mediated response (118). A mutually costimulatory interaction between DC and type I NKT cells ensues following encounter with CD1d/antigen complexes displayed by immature DCs. Ligation of APC-expressed CD40 with upregulated CD40L on type I NKT cells induces DCs’ maturation with higher surface expression of MHC class II, the costimulatory molecules CD40, CD80, CD86, CD70 and the endocytic receptor DEC205 and potent IL-12 production (119, 120). Sustained IL-12 secretion by mature DCs induces IFN-γ production by NKT cells (121–126). Mature DCs reciprocally enhance expression of CD40L and IL-12 receptor on type I NKT cells providing a strong feed forward signal that amplifies IFN-γ responses (119, 127). Ligation of chemokine receptor CXCR6 on the surface of type I NKT cells by its ligand CXCL16 expressed on APCs can also provide costimulatory signal resulting in robust α-GalCer-induced type I NKT activation (128, 129). α-GalCer-induced type I NKT cells can provide cognate licensing for cross-priming CD8 alpha ^+^ DCs to produce CCL17, which attracts CCR4^+^CD8^+^ T cells for subsequent activation (130, 131). Presence of phenotypic maturation ligands, suitable cytokines (IFN-γ), other functional immunostimulatory factors on type I NKT licensed DC can induce activation of CD8 T cells and their polarization toward antitumor effector function (119, 132–134). Release of various cytokines such as IL-2, IL-12, and IFN-γ by type I NKT cells leads to activation and expansion of NK cells into lymphokine-activated killer (LAK) cells. These LAK cells upregulate the effectors or adhesion molecules such as perforin, NKp44, granzymes, FasL, and TRAIL and secrete IFN-γ to adhere and lyse tumor cells (135, 136). Type I NKT cells can form bidirectional interactions with B cells, wherein B cells can present lipid antigens to type I NKT cells through CD1d (137) and NKT cells can license B cells to effectively prime and activate antitumor CTL responses (138, 139) and can also directly provide B cell help to enhance and sustain humoral response (57, 140–143).

## Altering the Effects of Immunosuppressive Cells in TME

Tumor-associated macrophages (TAMs) are prominent immunosuppressive immune cells present in the TME (144). TAMs contribute to tumor progression by enhancing angiogenesis, tumor cell invasion, suppression of NK, and T cell responses (145, 146). Type I NKT cells were found to co-localize with CD1d-expressing TAMs in neuroblastoma and kill TAMs in an IL-15 and CD1d-restricted manner (90, 147). Besides TAMs, type I NKTs can alter the effects of CD1d^+^ myeloid-derived suppressor cells (MDSCs)-mediated immune suppression. MDSCs are heterogeneous population of cells of myeloid origin, which often accumulate during tumor growth and contribute to immune escape and tumor progression (148). In a model of influenza A viral infection, adoptive transfer of type I NKTs inhibited arginase 1 and nitrous oxide synthase-mediated suppressive activity of MDSCs. The ability of type I NKT cells to abolish the suppressive activity of MDSCs was found to be dependent on CD1d and CD40 interactions (149). In a tumor model, α-GalCer-loaded MDSCs facilitate conversion of immature MDSCs to mature APCs capable of eliciting cytotoxic NK and T cell immune response against cancer cells (150). De Santo et al. reported type I NKT cell-mediated reversal of immunosuppressive activity of neutrophils in melanoma, serum amyloid A1 (SAA-1) derived as consequence of tumor-associated inflammation induced differentiation of IL-10-producing neutrophils causing suppression of antigen-specific T cell responses. Conversely, SAA-1 also enhanced CD1d-CD40 dependent interaction between the suppressive neutrophils and type I NKT cells. This crosstalk lead to dephosphorylation of Erk, p38, and phosphatidylinositol-3-OH kinase, which in turn lead to inhibition of IL-10 secretion and promotion of IL-12 production by neutrophils, reinstating the proliferation of antigen-specific CD8^+^ T cells (151).

## Suppression of Tumor Immunity by Type II NKT Cells

In contrast to the established protective role of type I NKT in most murine tumor models, type II NKT cells have been shown to possess a more suppressive/regulatory role in tumor immunity (4, 59, 65, 152). Comparison of antitumor response in Jα18-deficient mice (which lack only type I NKT) with CD1d deficient mice (which lack both type I and II NKT cell) revealed that type II NKT cells were responsible for suppression of antitumor responses in several murine tumor models (152–154). Furthermore, sulfatide-reactive type II NKT cells was shown to antagonize the protective antitumor immune responses mounted by α-GalCer-stimulated type I NKT cells (47). Sulfatide activated murine type II NKT cells were reported to inhibit proinflammatory functions of type I NKT cells, conventional T cells and DCs and also induce tolerization of myeloid DCs (155). A major attribute of type II NKT-mediated suppression of tumor immunity is elevated production of IL-13 and IL-4 cytokines capable of skewing the cytokine response predominantly toward tumor-promoting Th2 type. In a mouse model of transformed recurrent fibrosarcoma, type II NKT cells was shown to suppress cytotoxic T cells through IL-13 production *via* IL4R and STAT6 axis and also induce MDSCs producing immunosuppressive cytokine TGF-β (71). Similarly, LPC reactive type II NKT cells have been shown to preferentially produce IL-13 and exhibit immunoregulatory role in myeloma patients (58). Concentration of LPC, a phospholipid associated with inflammation, was found to be elevated in myeloma sera. Progressive myeloma disease is associated with a decline as well as dysfunctional activation of type I NKT cells and increased frequency of type II NKT cells (58, 78). The preferential production of IL-13, a cytokine implicated in promoting tumor growth, by LPC specific type II NKT cells suggests their role in disease progression (58). Recently, we have shown a possible implication of type II NKT cells in the development of B-cell malignancies associated with GD. GD is uniquely associated with increased cancer risk particularly with multiple myeloma (156). GD is a lysosomal storage disorder caused due to an inherited deficiency of the acidic β-glucosidase enzyme, resulting in marked accumulation of β-glucosylceramide (β GlcCer) and its deacylated product, glucosylsphingosine (LGL1). Increased frequency of LGL1-specific type II NKT cells with reduced frequency of type I NKT cells was observed in murine model and patients of GD. Interestingly, LGL1 reactive type II NKT cells demonstrated follicular helper T cell phenotype and were able to provide help to germinal center B cells to produce lipid-reactive antibodies (57). In both patients and mice with GD having monoclonal gammopathy, the monoclonal immunoglobulin was found to be reactive to Gaucher lipids (157). Though studies described earlier hint to pro- and antitumor functional dichotomy between type I and type II NKT, respectively, there are several emerging evidences challenging this paradigm, and the pro/antitumor roles of these cells may be context or activation-dependent. While type I NKT cells have been shown to assume immune-suppressive role in several tumor settings (158–161), a recent study showed that CpG-activated type II NKT cells secreted IFN-γ rather than IL-13, which in turn enhanced the activation and function of CD8^+^ T cells and contributed to the antitumor effect of CpG in the B16 melanoma model (162).

## Preclinical Studies

There are several theoretical advantages for harnessing type I NKT cells against cancer. NKT cell can simultaneously target both MHC positive and negative tumor cells due to ability to activate both antigen-specific CD8^+^ T cells and NK cells. Second, type I NKT cells show strong adjuvant activity thereby activating both innate and adaptive immune cells. Finally, NKT cells have the ability to convert immature and or tolerogenic DCs found in tumor bed into mature DCs capable of initiating tumor specific CD8^+^ T cell response. However, major limitations in targeting NKT cell for tumor treatment are the cancer-mediated reversible defect in the number and function of type I NKT cells (73, 74, 76–78, 80, 163, 164). Circulating type I NKT cell deficiency leads to decreased proliferation and IFN-γ production by type I NKT cells, consequently skewing immune response to a pro-tumor Th2 cytokine profile (73, 74, 76–78, 80, 163, 164). In line with this observation, reduced type I NKT cell frequency was shown to correlate with poor survival, while increased type I NKT cell numbers capable of making IFN-γ have positive prognostic value for survival in cancer patients (74, 80, 163–167). To restore the numbers and function of type I NKT cells in cancer patients and murine models, several approaches like administration of α-GalCer either alone or with IL-12, administration of APCs (DC or irradiated tumor cells) with α-GalCer, adoptive transfer of *ex vivo* expanded and/or activated type I NKT cells, and finally a combination of α-GalCer with antibodies or fusion proteins have been exploited. Data from numerous studies on variety of experimental and spontaneous murine tumor models have shown significant role for NKT cells in launching of powerful antitumor immune responses (Table 1).

**Table 1:** Preclinical studies on natural killer T (NKT) cell-targeted immunotherapeutics.

Therapy regimen	Murine model/cancer type	Outcome	Immunological response	Reference
**Injection of α-GalCer/IL-12**				
IL-12 injection	FBL-3 erythroleukemia, B16 melanoma	Inhibition of tumor growth and metastasis	NKT cell produced IL-12-mediated tumor rejection NKT cell-mediated direct cytotoxicity	(168)
α-GalCer (i.v.)	Colon 26 hepatic metastasis adenocarcinoma model	Regression of Colon 26 nodules Inhibition of tumor growth in liver	Activation of natural killer (NK) cells, T cells, and NK1^+^ T cells	(169)
α-GalCer (i.p.)	B16 melanoma cells	Prevented liver metastasis	NK cell-mediated killing	(170)
α-GalCer (i.v.)	Spontaneous liver metastasis of reticulum cell sarcoma (M5076)	Suppressed growth of established liver metastases, prolonged survival time	Increased IFN-γ and IL-12 production by liver NKT cells	(171)
α-GalCer + OVA (i.v.) or OCH + OVA (i.v.)	C57BL/6 mice s.c. injected with murine thymoma that express OVA	Slower growth of tumor up until 10 days followed by rapid regression	Induction of cytotoxic effector cells with potent antitumor activity	(172)
α-GalCer (i.v.) + IL-12 i.p.	BL6-B16 melanoma	Effective against metastatic tumor	NKT activation with induction of Th1 immunity and CD4^+^, CD8^+^ T cells, and B cells activation	(173)
α-GalCer (i.v.) + IL-12 i.p.	BL6-B16-HM melanoma	Prevention of tumor at early stages	NKT and NK activation	(174)
α-GalCer (i.p.) 2 μg single dose	B16-BL6 melanoma cells	Subcutaneous tumor growth and tumor-induced angiogenesis at early time points	IFN-γ-dependent inhibition of tumor angiogenesis by α-GalCer α-GalCer-activated NKT cells and secondarily activated NK cells contributed to the inhibition of endothelial cell proliferation *via* their IFN-γ production	(175)
α-GalCer (i.p.)	MCA induced sarcoma, mammary carcinomas in Her-2/neu transgenic mice, spontaneous sarcomas in p53^−/−^mice	Inhibition of primary tumor formation	NK cell and T cell activation Higher serum levels of IFN-γ and IL-4 TRAIL-dependent antimetastatic activity	(176)
α-GalCer (i.p.) + IL-12 i.p.	TRAMP prostate tumor	Reversion of prostrate tumor-mediated IFN-γ secretion by type I NKT cells	α-GalCer and IL-12 bypasses tumor cell-induced block of IFN-γ production	(91)
α-GalCer (i.v.) single dose	Mantle cell lymphoma	Inhibition of disease development Delayed disease progression	NKT activation	(177)
α-GalCer (i.p.) 2 μg	5T33 multiple myeloma	Significant reduction in micro vessel density	Possible role of IFN-γ from stimulated type I NKT cells in the antiangiogenic process	(178)
Priming with DNA vaccine expressing human papillomavirus type 16 E7^+^ α-GalCer and boosting with E7-pulsed DC-1	E7-expressing tumor model TC-1	Prolonged survival of vaccinated animals	E7-specific CD8^+^ T-cell responses	(179)
***Ex vivo*-generated dendritic cell (DC) loaded with α-GalCer/dying tumor cells**				
α-GalCer-loaded DC	B16 melanoma cells, LLC (lung metastatic model)	Inhibition of tumor metastasis in liver and lung Eradication of established tumor metastasis	Activation of NKT cells	(180)
α-GalCer-loaded ES DC genetically engineered to express a model antigen OVA + SLC/CCL21	MO4 (ova expressing melanoma)	Protection against tumor Enhanced antitumor activity, rejection of tumor cell	Synergic activation of antigen reactive CTL and α-GalCer-activated NKT cells	(181)
α-GalCer + CD4-hepatic NKT	MCA-induced sarcoma	Tumor regression	NA	(115)
α-GalCer-loaded irradiated tumor cells	A20 lymphoma, Meth A sarcoma, J558	Long-lived tumor immunity	Type I NKT, CD8^+^ T cells, CD4^+^ T cells contribute to tumor resistance Activation and proliferation of antigenic specific T cells Secretion of IFN-γ and IL-2	(182)
α-GalCer-loaded DC	Ductal pancreatic adenocarcinoma	Decrease in tumor growth and prolonged survival	Expansion of IFN-γ-producing NKT	(183)
α-GalCer-loaded tumor cell	A20 lymphoma	Tumor regression, resistance to tumor challenge	CD4^+^ T cells mediate antitumor activity	(184)
α-GalCer-loaded tumor cell	B16 melanoma cells, WEHI-3B myelomonocytic leukemia, EL4 thymoma tumor cells transfected with CD1d	Better survival with metastatic development thwarted	NKT and NK cell activation with induction of IFN-γ and IL-12p70 secretion	(185)
BM DC loaded with combination of tumor Ag and α-GalCer and anti-CD25 Ab	B16 melanoma cells	Delayed onset of tumor growth	Prolonged proliferative burst of responding CD8^+^ T cells	(186)
α-GalCer-loaded irradiated tumor cells	VK*Myc mice, AML-ET09G, Eu-myc lymphoma	Reduction in tumor load, resistance to rechallenge	Expansion NKT and NK cells IL-12-dependent IFN-γ production by NKT and NK cells	(187)
α-GalCer-loaded mature DC	5T33 multiple myeloma	Increased survival	Increased IFN-γ and Th1 response that tapers off at the end of disease	(178)
α-GalCer-loaded irradiated tumor cells	Multiple myeloma (MOPC315BM)	Retarded tumor growth Regression of established tumors Protection of surviving mice from tumor rechallenge	Expansion and activation of NKT cell *in vivo* Induction of strong myeloma specific antibodies and CD8^+^ CTL and memory T cells Decreased Treg frequency	(188)
α-GalCer delivery to CD8a^+^ DCs with anti-DEC205 decorated nanoparticles	B16 F10 melanoma cells expressing Ova	Potent antitumor responses	Type I NKT-mediated transactivation of NK cells, DCs, and gDT cells	(189)
α-GalCer-loaded irradiated tumor cells	C1498 leukemia model	Prevention of new leukemia development however no protective benefit in established leukemia	NKT cells are activated by langerin^+^CD8^+^ DC leading to generation of CD4^+^CD8^+^ T cells	(190)
α-GalCer loaded in CXCL16^hi^ BMDCs	B16 melanoma model	Inhibition of metastasis	Increased IFN-γ^+^ and Tbet^+^ type I NKT cells, enhanced serum IFN-γ levels	(191)
α-GalCer-loaded tumor cell + TLR9 agonist (CpG1826)	Colon cancer	Inhibition of established tumor Prolonged survival of tumor bearing mice Resistance to tumor rechallenge	Type I NKT activation and DC maturation IFN-γ secretion by type I NKT and NK cells Redirection of Th2 response toward Th1 immune response by DC produced IL-12	(192)
α-GalCer-loaded DCs + tumor cells	B-cell lymphoma	Potent long-lasting tumor-specific antitumor immune response	Type I NKT cells secreting IFN-γ T cells and NK cell-mediated antitumor effect	(193)
**Adoptive transfer of *ex vivo*-expanded NKT cells**				
IL-12-activated NKT i.v. injection (4 times)	B16 melanoma cells	Inhibition of tumor metastasis	Strong cytotoxic activity by activated NKT on metastasized tumor cells in liver	(194)
*In vitro*-expanded CD8^+^ NKT cells redirected with humanized bispecific antibody F(ab′)2HER2xCD3	HER2-expressing ovarian carcinoma	Rapid tumor regression with prolonged survival	High efficacy of target cell killing by CD8^+^ NKT	(195)
α-GalCer + *ex vivo*-expanded NKT	C1R B-cell lymphoblasts	Reduced growth of CD1d^+^ leukemic cells and eradication of neoplastic clone	NKT cell-mediated cytotoxicity on CD1d^+^ nodules Presence of NKT cells infiltrating lymphoid nodules	(196)
Tumor-sensitive T cells + CD25^+^ NKT cells + epigenetic drug decitabine	Carcinoma	Prolonged survival of animals bearing metastatic tumor cells	Decitabine functioned to induce the expression of highly immunogenic cancer testis antigens in the tumor, while also reducing the frequency of myeloid-derived suppressor cells (MDSCs) The presence of CD25^+^ NKT cells rendered T cells resistant to remaining MDSCs	(197)
**Monoclonal antibodies stimulating NKT and α-GalCer with fusion proteins**				
Anti-CD1d mAbs	4T1 mammary carcinoma, R331 renal carcinoma and CT26L5 colon adenocarcinoma	Suppression of established tumor growth	Activation of CD1d^+^ antigen-presenting cell to produce tumor inhibiting IFN-γ and IL-12 Blocking of type II NKT cells activity in these models	(198)
Combination mAbs anti-DR5^+^ CD137^+^CD1d (1DMab)	4T1 mammary carcinoma, R331 renal carcinoma, and CT26L5 colon adenocarcinoma	Suppression and or eradication of established tumors	Tumor rejection was dependent on CD8^+^ T cells, IFN-γ, and CD1d and partially dependent on NK cells and IL-12	(199)
α-GalCer-loaded recombinant soluble (sCD1d) + HER2-specific scFv antibody fragment	HER2-expressing B16 melanoma model	Potent inhibition of lung metastasis	Specific localization to tumor site and accumulation of type I NKT, NK, and T cells at tumor site	(200)
α-GalCer-loaded sCD1d fusion proteins	MC38 colon carcinoma transfected with human CEA	Inhibition of tumor growth	Strong and prolonged reactivity of type I NKT cells IFN-γ production by NK and NKT cells Direct lysis by NKT cells	(201)
**Type I NKT chimeric antigen receptor (CAR)**				
CAR.GD2 NKT with CD28, 4-1BB	Metastatic neuroblastoma	Potent antitumor activity and long-term survival	Potent dose dependent cytotoxicity against GD2-positive neuroblasts Enhanced *in vivo* persistence of NKT cells with systemic elevation of Th1 cytokines Effective localization to tumor site without inducing GVHD	(202)
CD62L^+^CAR.CD19 NKT	B-cell lymphoma	Prolonged survival of tumor bearing mice and sustained tumor regression	CD62L^+^ NKTs have prolonged persistence *in vivo*	(203)

Type I NKT cells were shown to be indispensable in mediating IL-12-mediated antitumor effects in low- and moderate-dose IL-12 treatment models (91, 169, 204). IL-12 was found to activate the NKT cell-mediated lysis of tumor cells and also induce IFN-γ production by type I NKT cells. Administration of soluble α-GalCer leads to activation and expansion of type I NKT cells, creating a milieu of immune-stimulatory cytokines including IFN-γ and costimulatory molecules, resulting in maturation of host APC thus enhancing antitumor T cell response. IFN-γ production by type I NKT cell was found to be pivotal in inducing NK cell activation, proliferation of memory CD4^+^ and CD8^+^ T cell effector functions, and inhibiting angiogenesis, all contributing to effective immune response against tumor. One of the major drawbacks of administering soluble free α-GalCer is that it causes type I NKT cell to adopt an anergic state causing unresponsiveness to sequential stimulation with α-GalCer (205). To circumvent this problem, mice were administrated DCs loaded with either α-GalCer alone or in combination with tumor antigens (180, 182, 187, 190, 206). α-GalCer-pulsed APCs induced a more prolonged cytokine response as well as powerful antitumor immune response than α-Galcer alone (180, 207). Another recent immunotherapeutic approach has been to load autologous irradiated tumors, which act as source of tumor antigens with α-GalCer (121, 182, 187, 188). A big improvement of this approach is CD1d-mediated cross-presentation of endogenous glycolipids and or α-GalCer from tumor cells to NKT cells, leading to DC maturation and consequently effective long-term T cell resistance to the tumor (128). Another approach involved adoptive transfer of *ex vivo* expanded and or activated type I NKT cells to restore type I NKT cell numbers in preclinical models of melanoma and lymphoid neoplasms (194, 196, 208). This approach has been shown to be more effective compared to the i.v. administration of α-GalCer (194). Finally, combination therapy using monoclonal Abs targeting CD1d alone or in combination with tumor cell death inducing and immunomodulating mAbs has emerged as promising immunotherapeutic candidate against CD1d-negative cancers (199). Stirnemann and Corgnac et al. attempted to target α-GalCer to tumor site by using constructs consisting of either α-GalCer/CD1d molecules alone or fused to tumor Ag specific scFv fragments in a colon carcinoma and murine melanoma model, respectively, and reported specific tumor localization of type I NKT activating potent antitumor responses compared to α-GalCer alone (200, 201). Preclinical studies obtained using chimeric antigen receptors (CARs) with engineered type I NKT cells have yielded promising result. CAR-bearing type I NKT cells effectively localized to the tumor sites, eliminating tumor cells, and exhibited potent and specific cytotoxicity against TAMs without producing graft-versus-host disease (202). Recently, CD62L^+^CD19^−^specific CAR-engineered NKT cells have been shown to possess superior therapeutic activity in a B-cell lymphoma model (203).

## Clinical Trials of NKT Cells

Based on the preponderance of data from preclinical mice models, showing that activation of type I NKT cells plays a substantial role in providing protection against tumor growth and metastasis of several tumors, different clinical trials have been initiated to harness NKT cell’s antitumor potential (Table 2). However, while direct administration of soluble α-GalCer in cancer patients was well tolerated, it failed to yield any clinical response (209). Potential reasons for the low efficacy in human trials could be attributed to insufficient drug delivery, inter-individual variability and very low type I NKT cell numbers at baseline, induction of anergy or regulatory IL-10-producing type I NKT cells (205, 210, 211). To overcome these limitations of soluble α-GalCer administration and improve NKT-mediated antitumor responses, multiple clinical trials were performed using autologous α-GalCer-pulsed APCs in patients with advanced and recurrent non-small cell lung cancer, head and neck squamous cell carcinoma (Table 2). Different types of APCs and alternative routes to efficiently target activated NKT cells directly to cancer region were optimized to achieve objective antitumor responses. Though promising, this strategy too suffers from certain caveats like the treatment is again dependent on the baseline NKT levels, which are inevitably low in most cancer patients. Second, it is difficult to obtain large number of autologous monocyte-derived DCs (moDCs) from immune suppressed cancer patients and also cumbersome for *ex vivo* generation of DCs in compliance with good manufacturing practices regulations. Another strategy involves adoptive transfer of *in vitro*-expanded autologous type I NKT populations. Clinical trials using this approach in non-small cell lung cancer and advanced melanoma do show increase in type I NKT expansion and elevated serum IFN-γ levels *in vivo*; however, further optimization of the protocols and perhaps combination approaches such as combining with immune checkpoint blockade may be needed to obtain a significant clinical response. Remarkably, combining activated type I NKT cells and α-GalCer-pulsed APCs has been reported to enhance the low antitumor response observed with monotherapy employing either NKT or APCs alone in head and neck squamous cell carcinoma patients (212, 213). Similarly, combining regimen of α-GalCer-pulsed DCs and the immune-modulatory drug lenalidomide in treating multiple myeloma patients leads to type I NKT expansion with downstream activation of NK, monocytes and decrease in tumor-associated M spikes (214).

**Table 2:** Clinical studies using natural killer T (NKT) cell-targeted immunotherapeutics.

Treatment	Injection site, number of injections/cycles	Tumor type	Number of patients	Safety	Clinical outcome	Immunological response	Reference
**Direct α-GalCer injection**							
α-GalCer	i.v., 50–4,800 µg/m^2^; 3 days 4 weekly cycle	Solid tumors	24	No dose limiting toxicity	7/24 patient stable disease for 123 days No clinical response	Transient decrease in type I NKT and natural killer (NK) cells from circulation Increased serum cytokine levels of IFN-γ and GM-CSF in 5/24 patients Cytotoxicity in 7/24 patients. The effect was dependent on pretreatment type I NKT cell numbers.	(209)
***Ex vivo*-generated dendritic cell (DC) pulsed with α-GalCer**							
α-GalCer-pulsed CD1d-expressing immature monocyte-derived DCs (moDCs)	i.v., 2 doses over 2-week cycle	Metastatic malignancy	12	No severe toxicity	2/12 patients had decreased serum tumor markers 1 subject developed extensive necrosis of tumor-infiltrating bone marrow 2 patients with hepatic infiltration had reduction in serum hepatocellular enzyme levels. Clinically apparent treatment specific inflammatory response at tumor sites	NKT cell, T cell activation Increase in NK cell numbers, activation and enhanced cytotoxicity Increased IFN-γ (10/10) and IL-12 (6/9) levels in serum	(215)
α-GalCer-pulsed IL-2/GM-CSF cultured PBMCs	i.v., 4 doses, 5 × 10^7^ cells (level 1) 5 patients, 2.5 × 10^8^ cells (level 2) 3 patients, 3 × 10^9^ cells (level 3) 3 patients	Non-small cell lung cancer	11	No severe toxicity	Stable disease in 3 patients	Expansion of type I NKT cells in 3/11 patients Elevated IFN-γ mRNA levels in 1/11 patients	(216)
α-GalCer-pulsed immature moDCs	i.v., 4 injections of 1 × 10^9^ cells	Non-small cell lung cancer	17	No severe toxicity	Stable disease in 5 patients, median survival time 18.6 months	Expansion of type I NKT cells in 16/17 patients Elevated IFN-γ-producing cells by ELISPOT in 10/17 patients	(217)
α-GalCer-pulsed immature moDCs	4 treatments total with iv., 2 treatments, and intradermal (i.d.) 2 treatments, doses ranging from 5 × 10^5^, 5 × 10^6^, and 2–5 × 10^7^ cells	Metastatic solid tumor	12	Safe and well tolerated	Stable disease in 6/10 patients 3 patients show minor objective defined as reduction in tumor mass/marker 9/12 had transient therapy related tumor inflammation	Dose of 5 × 10^6^ *via* i.v. route gave the most reproducible result of NKT activation resulting in increased circulating type I NKT cells levels with NK and T cell activation and increased serum IFN-γ levels	(218)
α-GalCer-pulsed IL-2/GM-CSF cultured PBMCs	i.v., 1 injection	Non-small cell lung cancer	4	No serious toxicity	NA	Increased mobilization of type I NKT cells into primary site of the lung cancer Augmented IFN-γ-producing ability of tumor-infiltrating type I NKT cells	(219)
α-GalCer-pulsed antigen-presenting cell (APCs)	Nasal sub-mucosal injections, 2 treatments with 1-week interval	Head and neck squamous cell carcinoma	9	Safe and well tolerated	1 patient showed partial response, 7 patients showed stable disease	Increase in circulating type I NKT numbers (4/9) Expansion of α-GalCer reactive IFN-γ-producing cells in PBMCs (8/9)	(220)
α-GalCer-pulsed mature moDCs	i.v. 2 injections	Advanced cancer	5	Safe and well tolerated	Patients had stable disease. 3 patients had decreased M spike levels in serum and urine	>100-fold expansion of type I NKT cell subsets sustained up to 5 months after vaccination Type I NKT cell activation was associated with increased serum levels of IL-12p40, IP-10, and MIP-1β	(221)
**Adoptive transfer of autologous *ex vivo*-expanded NKT cells**							
*Ex vivo*-expanded NKT cells with autologous α-GalCer-pulsed PBMCs	i.v., 2 doses, 1 × 10^7^ cells (level 1) 6 patients, 2.5 × 10^7^ cells (level 2) 3 patients	Non-small cell lung cancer	9	No adverse effects	No tumor regression Stable disease in 2/9 patients	Absolute number of circulating type I NKT cells increased in 2/3 case receiving level 2 dose IFN-γ production augmented in all 3 cases receiving level 2 dose	(222)
*Ex vivo*-expanded NKT cells	i.v., 3 infusions of 25 × 10^7^ cells/infusion spaced 2 weeks apart with pretreatment of GM-CSF before cycle 2 and 3 to enhance DC functions	Advanced melanoma	9	No adverse effects	Patients deceased (3/9) Patients progressed (3/9). Median follow-up for 63 months	Type I NKT infusions appeared to cause transient peak of circulating type I NKT cells that were enhanced by GM-CSF pretreatment Increased number of activated monocytes Elevated IFN-γ production (5/8)	(208)
**Combination therapies**							
*Ex vivo*-expanded NKT cells (intra-arterial) and autologous α-GalCer-pulsed PBMCs (*via* nasal submucosal)	1 × 10^8^ α-GalCer-loaded APCs submucosal injections (2 injections) followed by *in vitro* activated type I NKT cells (i.a) into tumor feeding artery (1 injection)	Head and neck squamous cell carcinoma	8	Serious adverse event (1). Mild adverse events (7)	Partial response (3/8) Stable disease (4/8) Progressive disease (1/8)	Increase in circulating type I NKT numbers (6/8) Expansion of α-GalCer reactive IFN-γ-producing cells in PBMCs (7/8)	(212)
*Ex vivo*-expanded NKT cells (intra-arterial) and autologous α-GalCer-pulsed PBMCs (*via* nasal submucosal)	1 × 10^8^ α-GalCer-loaded APCs submucosal injections (1 injection) followed by *in vitro* activated type I NKT cells (i.a) into tumor feeding artery (1 injection)	Head and neck squamous cell carcinoma	10	No adverse effects	Objective tumor regression (5/10) Stable disease (5/10) Antitumor effects (8/10)	Expansion of type I NKT in PBMC (7/10) and TIL correlating with partial response (6/6) Elevated expansion of IFN-γ spot forming cells in PBMCs (8/10) and in tumor tissue	(213)
α-GalCer-pulsed mature moDCs + LEN	i.v., LEN (oral 10 mg/day), 28 day ×3 cycles	Multiple myeloma	6	Safe and well tolerated	3/4 patients show reduction in tumor-associated M spike after therapy	Activation of NKT, NK, monocyte, and eosinophils	(214)

## Emerging Approaches

### Adoptive Transfer of Type I NKT Cells

Advanced cancer patients with low NKT cell numbers may benefit from development of *in vitro* methods for generation of large numbers of functional NKT cells which can be further used for adoptive transfers. NKT cells have been generated from CD34^+^ cells isolated from cord blood using IL-15 and stem cell factor (flt-3 ligand) in liquid culture system. Watarai et al. successfully differentiated murine induced pluripotent stem cells (iPSCs) into functional NKT cells *in vitro* that secreted large amounts of Th1 cytokine IFN-γ acting as adjuvant and antitumor agent (223). Recently, protocol to generate human type I NKT cells *in vitro* from iPSC that are competent in eliciting antitumor activity has been generated (224). Human type I NKT cells can also be reprogrammed to pluripotency followed by redifferentiation back to type I NKT cells *in vitro* using an IL-7/IL-15-based cytokine combination (225). The immunological features of re-differentiated type I NKT cells and their unlimited availability from iPSCs offer a potentially effective immunotherapy against cancer. Functionally mature human NKT cells have been also generated from bone marrow-derived adult hematopoietic stem-progenitor cells by expansion with CD1d-Ig-based artificial-presenting cells (226). Owing to the feasibility of producing large quantities of competent NKT cells, stem cell-derived type I NKT cells offer a promising strategy for effective anticancer immunotherapy.

## Alternate Ligands

As discussed earlier, while α-GalCer is a potent activator of type I NKT cells, α-GalCer suffers from few drawback that limits its use as effective cancer immunotherapeutic. For example, α-GalCer induces anergy in type I NKT cells. This has led to preclinical exploration of several alternate ligands that are now poised to enter the clinic. Synthetic glycolipids or α-GalCer analogs chemically modified to induce more precise and predictable cytokine profile than α-GalCer have been synthesized and tested. These analogs as compared α-GalCer, show superior anticancer immunity in tumor mouse models and therefore hold great potential as an alternative vaccine adjuvant (227–229). As compared to α-GalCer, alternative non-glycosidic type I NKT-cell agonist threitol ceramide promoted stronger activation of human and mouse type I NKT cells and stronger antitumor responses in comparison to α-GalCer, making it potential candidate for NKT cell-based clinical trials (230). Another interesting prospect is encapsulating α-GalCer or other lipids in nanoparticle carriers or liposomes decorated with Abs or ligands to target specific APCs. These approaches have several advantages like slower release of α-GalCer, specific targeting of APC subset, lower amounts of α-GalCer required to activate NKT cells than soluble α-GalCer (231). Positive therapeutic effect of α-GalCer-loaded octa-arginine modified liposomes was reported in melanoma murine model (232). Administration of α-GalCer and ovalbumin coencapsulated PLGA nanoparticles provided significant prophylactic and therapeutic responses in mouse melanoma model by enhancing activation and tumor infiltration of the antigen-specific CD8^+^ T cell (233).

## Combination Approaches

A major limitation of the initial studies targeting NKT cells in cancer is that these studies were conducted using single agent strategies and did not account for blockade of immune checkpoints or other immune-suppressive factors. PD-1:PD-L pathway has been shown to play an important role in mediating αGalCer-induced anergy in NKT cells. Antibody-mediated blockade of PD-1:PD-L interactions at the time of α-GalCer treatment prevent the induction of type I NKT anergy and also enhance the antitumor activities of αGalCer. Therefore, combination of NKT-targeted therapies with PD-1:PD-L blockade should be considered (234). Synthetic lipopeptide vaccines based on conjugation of MHC-binding peptide epitopes to α-GalCer displayed promising antitumor activity in a melanoma model. The principle behind these vaccines is to simultaneously provide both adjuvant and antigen to the same cell in a controlled fashion. Application of this vaccine technology using different tumor antigens might serve as a novel strategy for diverse malignancies (235). Combination of type I NKT-targeted DC vaccine with low dose of lenalidomide led to promising clinical activity in myeloma (214). Therefore, there is an unmet need to pursue combination approaches targeting type I NKT cells to better harness the antitumor properties of type I NKT cells in the clinic.

## Concluding Remarks

Natural killer T cells are an important component of the TME and play key roles in regulating antitumor immunity. Although preclinical studies with NKT cell-targeted therapies in murine tumor models have been positive, clinical translation of these results has proven challenging. Translational challenge could be attributed to incomplete knowledge of human NKT subsets. Generation of improved preclinical models that replicate human NKT cell response is needed to gain insights into the cross talk between APCs and NKT subsets and to improve the efficacy of NKT cell-targeting therapies.

## Author Contributions

Both SN and MVD participated in conceptualization and drafting of the article as well as critical revision of the article for important intellectual content. Both authors gave final approval of the submitted publication.

## Conflict of Interest Statement

The authors declare that the research was conducted in the absence of any commercial or financial relationships that could be construed as a potential conflict of interest.
